# Significance of CT attenuation and F-18 fluorodeoxyglucose uptake of visceral adipose tissue for predicting survival in gastric cancer patients after curative surgical resection

**DOI:** 10.1007/s10120-019-01001-2

**Published:** 2019-09-04

**Authors:** Jeong Won Lee, Myoung Won Son, Il Kwon Chung, Young Sin Cho, Moon-Soo Lee, Sang Mi Lee

**Affiliations:** 1 Department of Nuclear Medicine, Catholic Kwandong University College of Medicine, International St. Mary’s Hospital, Incheon, Korea; 2 Department of Surgery, Soonchunhyang University Cheonan Hospital, 31 Suncheonhyang 6-gil, Dongnam-gu, Cheonan, Chungcheongnam-do 31151 Korea; 3 Division of Gastroenterology, Department of Internal Medicine, Soonchunhyang University Cheonan Hospital, Cheonan, Korea; 4 Department of Nuclear Medicine, Soonchunhyang University Cheonan Hospital, 31 Suncheonhyang 6-gil, Dongnam-gu, Cheonan, Chungcheongnam-do 31151 Korea

**Keywords:** Gastric cancer, Fluorodeoxyglucose F18, Positron emission tomography, Prognosis, Visceral adipose tissue

## Abstract

**Background:**

The purpose of this study was to investigate the prognostic significance of computed tomography (CT) attenuation and F-18 fluorodeoxyglucose (FDG) uptake of visceral adipose tissue (VAT) to predict peritoneal recurrence-free survival (RFS) as well as RFS and overall survival (OS) in patients with advanced gastric cancer (AGC).

**Methods:**

We retrospectively enrolled 117 patients with AGC who underwent staging FDG positron emission tomography (PET)/CT and subsequent curative surgical resection. CT attenuation and FDG uptake (SUV) of VAT and maximum FDG uptake of primary tumor (SUVmaxT) were measured from PET/CT images. The relationship of VAT attenuation and SUV with clinico-histopathologic factors and survival was assessed.

**Results:**

There was a significant positive correlation between VAT attenuation and SUV (*p* < 0.001, *r* = 0.799). In correlation analyses, both VAT attenuation and SUV showed significant positive correlations with T stage, TNM stage, tumor size, and platelet-to-lymphocyte ratio (*p* < 0.05), and patients who experienced recurrence during the first 3-year after surgery had significantly higher VAT attenuation and SUV than those who had no recurrence (*p* < 0.05). Patients with high VAT attenuation and SUV showed significantly worse RFS, peritoneal RFS, and OS than those with low values (*p* < 0.05). On multivariate survival analysis, VAT attenuation was significantly associated with peritoneal RFS and OS and VAT SUV was significantly associated with OS (*p* < 0.05).

**Conclusions:**

CT attenuation and FDG uptake of VAT on staging FDG PET/CT were correlated with tumor characteristics and were significant predictive factors for peritoneal RFS and OS in patients with AGC.

## Introduction

The only curative treatment for advanced gastric cancer (AGC) is compete resection of the primary tumor with lymph node dissection [1]. However, recurrence occurred in up to 53.1% of AGC patients even after curative surgery, which usually leads to death [2–4]. Among the various recurrence patterns of gastric cancer, peritoneal recurrence is the most frequent pattern, comprising 45.9–58.8% of patients with recurrence [2–4]. Peritoneal recurrence is known to be associated with poor prognosis, showing median survival of less than 10 months [4–6]. Further, a previous study even revealed that patients with peritoneal recurrence had worse prognosis than those with distant recurrence, but, no peritoneal recurrence [4]. In previous studies, depth of tumor invasion, lymph node metastasis, Lauren classification, and Bormann classification have been shown to be independent predictors of peritoneal recurrence [2–5]. Recently, an attempt has been made to control peritoneal metastasis by intraperitoneal chemotherapy in patients at high risk of peritoneal recurrence [7]. Therefore, identifying a predictive factor for peritoneal recurrence might have profound significance in selecting candidates to such a treatment.

Adipose tissue is one of the main compositions of body mass and is comprised of heterogeneous cell populations including mature adipocytes, pre-adipocytes, macrophages, and fibroblasts [8]. Over the last decade, there has been growing evidence that adipose tissue acts as a real organ in metabolic and endocrine functions in addition to its known traditional role, regulation of energy homeostasis [8, 9]. Several recent studies have shown that dysfunctional adipocytes in inflammatory and hypoxic conditions can contribute to progression, invasion, and metastasis of cancer cells by secreting multiple adipokines, sustaining inflammatory condition, promoting angiogenesis, and providing energy to tumor cells [8, 10]. Furthermore, dysfunctional adipocytes interacting with cancer cells also induce inflammatory and fibrotic changes in adipose tissue [8, 11]. In previous clinical studies with imaging examinations, two imaging parameters were used to assess these qualitative changes in adipose tissue: CT attenuation measured on unenhanced computed tomography (CT) and F-18 fluorodeoxyglucose (FDG) uptake measured on positron emission tomography (PET) [12]. These imaging parameters of adipose tissue were shown to be associated with tumor stage and clinical outcomes in patients with diverse malignant diseases, suggesting that these parameters can reflect the adipose tissue microenvironment which promotes cancer cell growth and invasion [12–14]. Considering that gastric cancer develops and grows in an adipose tissue-dominated microenvironment [10], qualitative features of visceral adipose tissue (VAT) could also have significant association with characteristics and progression of gastric cancer. In cell culture studies, interaction between gastric cancer cells and adipocytes affected chemosensitivity of cancer cells and promoted metastasis to the peritoneum [15, 16]. However, the clinical significance of VAT imaging parameters in gastric cancer patients has not been reported.

In the present study, we measured CT attenuation and FDG uptake of VAT on FDG PET/CT images and evaluated whether these imaging parameters of VAT had a significant association with peritoneal recurrence-free survival (RFS), RFS, and overall survival (OS) in patients with AGC after curative surgical resection.

## Materials and methods

### Patients

This study was approved by the Institutional Review Board of our university. Because of the retrospective nature of the study, the requirement to obtain informed consent was waived. We retrospectively reviewed the medical records of patients who were diagnosed with gastric cancer and underwent surgical resection of the gastric cancer at our medical center between March 2012 and December 2016. Of these patients, 117 patients were finally enrolled in the study according to the following inclusion criteria: patients who (1) were histopathologically diagnosed with AGC (pT2-T4), (2) underwent pre-operative FDG PET/CT, and (3) subsequently underwent curative surgical resection of gastric cancer. We excluded patients (1) who were diagnosed with early gastric cancer (pT1) (*n* = 195), (2) who had distant metastatic lesions on staging work-up (*n* = 37), (3) in whom microscopic or macroscopic peritoneal seeding metastases were found during surgical exploration or malignant cells were found on peritoneal washing cytology (*n* = 9), (4) who had a previous history of malignancy or major abdominal surgery (*n* = 5), (5) who had CT images that were in appropriate for analyzing VAT (*n* = 3), and (6) who were not followed up for 2 years after surgery without event (*n* = 8). We also excluded two patients who had a rare histopathological type of gastric cancer (adenosquamous cell carcinoma).

All patients in the study underwent pre-operative examinations including physical examination, gastroduodenoscopy, contrast-enhanced abdominopelvic CT scan, FDG PET/CT scan, and blood test. Afterwards, curative surgical resection, subtotal or total gastrectomy with at least D2 lymph node dissection, was performed according to the treatment guidelines of the Japanese Gastric Cancer Association [1]. The median interval between FDG PET/CT and operation was 2.0 days (range 1.0–21.0 days). With surgical specimens, histopathological stages of all patients were assessed in accordance with the seventh edition of the American Joint Committee on Cancer staging guidelines. Gastric cancers were categorized into five histopathological subtypes: papillary adenocarcinoma, well-differentiated and moderately differentiated tubular adenocarcinoma, poorly differentiated adenocarcinoma, signet-ring cell carcinoma, and mucinous carcinoma [17]. Furthermore, all tumors were classified into two microscopic growth types according to the Lauren classification: intestinal and non-intestinal types. Diffuse, mixed, and non-classifiable types were included in the non-intestinal types [18]. According to the results of histopathologic assessment, adjuvant chemotherapy after surgery was recommended for patients who were diagnosed with TNM stage II and III. For adjuvant chemotherapy, capecitabine plus oxaliplatin (XELOX), TS-1 plus cisplatin, 5-fluorouracil plus cisplatin, or 5-fluorouracil plus oxaliplatin (FOLFOX) were performed. After curative surgery, follow-up examinations including gastroduodenoscopy, contrast-enhanced abdominopelvic CT scan, and blood tests (including serum carcinoembryonic antigen and cancer antigen 19–9) were performed every 6–8 months in the first 3 years and, subsequently, every 10–12 months. For patients who received adjuvant treatment, follow-up examinations were performed mid-chemotherapy and after completion of the adjuvant treatment. Afterwards, those patients with adjuvant treatment underwent routine follow-up programs. Cases with cancer recurrence during follow-up were categorized into three groups according to the site of cancer recurrence: locoregional recurrence (remnant stomach, site of anastomosis, gastric bed, regional lymph node, or peritoneum around the surgical site), peritoneal recurrence (peritoneal seeding nodule, increased peritoneal density, massive ascites, abnormally increased intestinal wall thickness, peribiliary infiltration, or Krukenberg tumor) and distant recurrence (distant lymph node and organ metastasis) [2].

### FDG PET/CT scan and image analysis

All FDG PET/CT scans were performed using a Biograph mCT 128 scanner (Siemens Healthcare, Knoxville, TN, United States). Before FDG PET/CT scan, all patients were instructed to fast for at least 6 h and only patients who showed blood glucose level of less than 200 mg/dL before FDG administration underwent PET/CT scanning. Sixty minutes after the intravenous injection of FDG at a dose of approximately 4.07 MBq/kg, FDG PET/CT scanning was performed from the skull base to the proximal thigh. Non-contrast-enhanced CT scan was initially performed for attenuation correction at 100 mA and 120 kV_p_. Subsequently, PET scanning was performed at 1.5 min per bed position in the three-dimensional acquisition mode. PET images were reconstructed with a point-spread-function based Gauss and Allpass filter algorithm and time-of-flight reconstruction with attenuation correction.

**Fig. 1:**
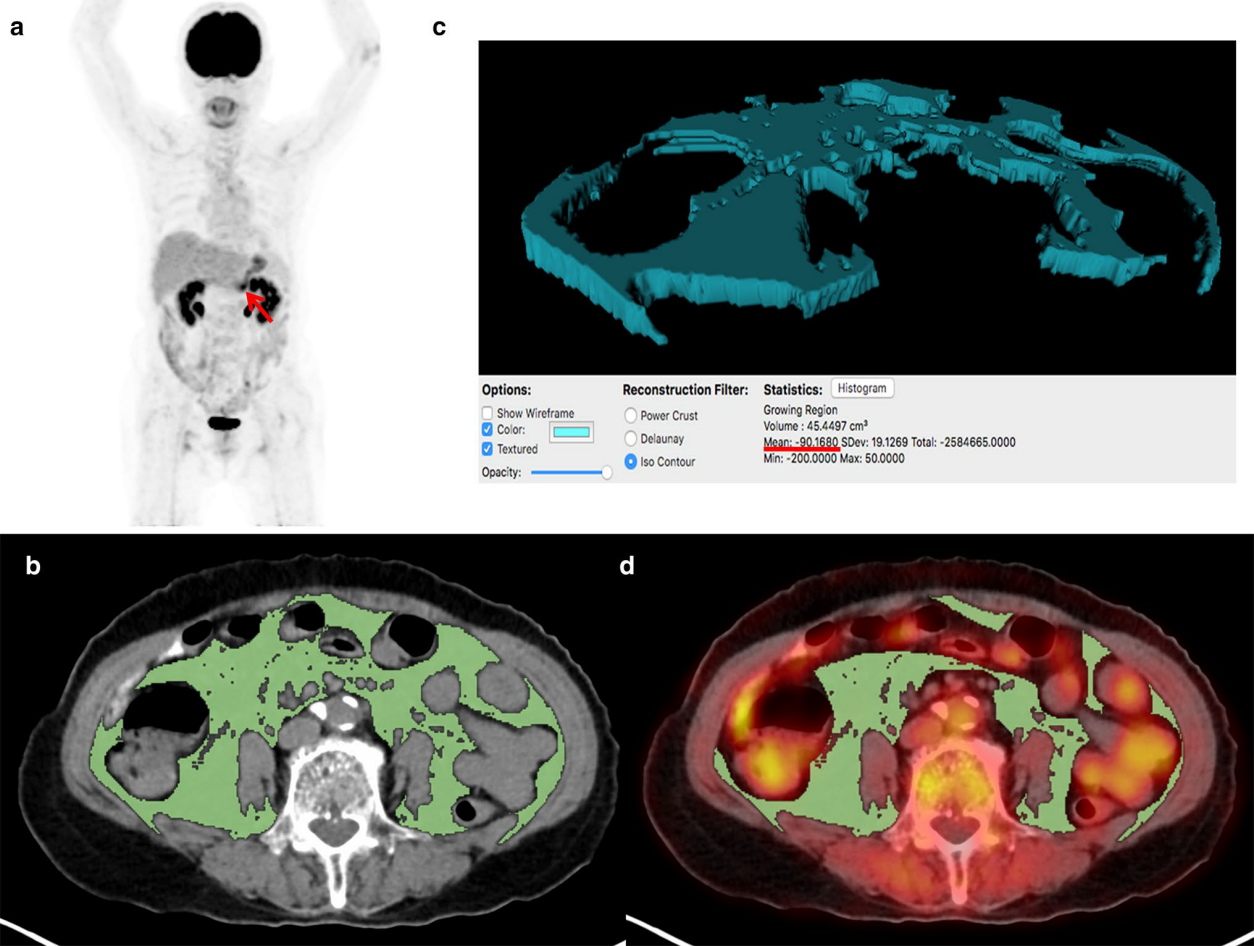
Measurement example of CT attenuation and SUV of VAT. A 79-year-old woman underwent FDG PET/CT for staging work-up of AGC, showing focally increased FDG uptake at lower body of the stomach on maximal intensity projection image (arrow) with SUVmaxT of 4.12 (**a**). On three consecutive transaxial CT images at the L4-5 spine level, the area of VAT defined as an intra-abdominal area with CT attenuation range between − 200 and − 50 HU was automatically delineated using OsiriX MD 10.0 software (**b**). With the areas of VAT on CT images, three-dimensional structure of VAT was automatically created and mean attenuation of the structure was calculated, which was − 90.17 (**c**). Afterwards, the VAT areas on CT images were exported to the corresponding fused PET/CT images. After the removal of FDG activity of the urine, bowels, and vessels, mean SUV of the areas was measured, and was found to be 0.49 (**d**). The patient underwent subtotal gastrectomy and was diagnosed with pT2N1 stage tubular adenocarcinoma. The patient experienced peritoneal recurrence 10.4 months after the surgery and died 13.3 months after operation

Two board-certified nuclear medicine physicians retrospectively evaluated FDG PET/CT images using a United States Food and Drug Administration-approved medical image viewer, OsiriX MD 10.0 software (Pixmeo, Geneva, Switzerland) without knowing clinical outcomes of the patients. First, FDG uptake of primary gastric cancer lesions was measured. A volume of interest (VOI) was manually drawn over the primary cancer lesion on transaxial PET/CT images and the maximum standardized uptake value (SUV) of primary cancer lesion (SUVmaxT) was measured. SUV was calculated as [decay corrected activity (kBq) per tissue volume (mL)]/[injected FDG activity (kBq) per body mass (g)]. In patients who had gastric cancer lesions with no discernibly increased FDG uptake, VOI was drawn in accordance with the tumor location seen on contrast-enhanced abdominopelvic CT images and gastroduodenoscopy. Afterwards, CT attenuation and FDG uptake of VAT on PET/CT images were measured (Fig. 1). To avoid the influence of the primary cancer lesion, three consecutive slices of the CT images at the level of the L4–L5 vertebrae, which is remote from the gastric cancer lesion, were selected for image analysis. VAT area, which is defined as an area with a CT attenuation range between − 200 and − 50 Hounsfield units (HU) in the intra-abdominal fat tissue, on those three slices of CT images was automatically identified (Fig. 1b) [12, 19]. Mean value of CT attenuation of the VAT area was measured (Fig. 1c). The VAT area on CT images was then exported to the corresponding PET images to measure FDG uptake of VAT. Physiologic FDG activities in the urine, bowels, and vessels that could affect the measurement of FDG uptake of VAT due to bowel movement during PET/CT scanning as well as the spillover FDG activity were manually removed. The mean SUV of the VAT area was then measured (Fig. 1d).

### Statistical analysis

To assess the reproducibility of measurement of VAT attenuation and SUV between the two readers, concordance correlation coefficients for VAT parameters were calculated. From the blood tests results obtained at staging work-up, the neutrophil-to-lymphocyte ratio (NLR) and platelet-to-lymphocyte ratio (PLR) were calculated for each patient. The Komogorov–Smirnov test was performed to evaluate the normality of distribution for continuous variables. Pearson’s correlation coefficients were calculated to evaluate the relationship between VAT parameters and other continuous variables. The Kruskal–Wallis test and Mann–Whitney *U* test were performed to evaluate differences of VAT parameters between patient groups. For survival analysis, the significance of the predictive value of each variable for RFS, peritoneal RFS, and OS was investigated using Cox proportional hazards regression test for univariate and multivariate analyses. Bonferroni adjustment was applied to correct for multiple testing. Survival time was defined as time from the day of curative surgical resection to the day of recurrence, peritoneal recurrence, or death. Patients who had no recurrence or survived were censored at the day of the last follow-up visit at our medical center. For continuous variables, optimal cut-off values were selected from the receiver-operating-characteristic (ROC) curve analysis, and all continuous variables were categorized into two groups according to those cut-off values. Survival curves for VAT parameters were estimated using the Kaplan–Meier method to calculate cumulative RFS, peritoneal RFS, and OS. All statistical analyses were performed using MedCalc Statistical Software version 18.10.2 (MedCalc software bvba, Ostend, Belgium). A *p* value of less than 0.05 was considered statistically significant.

## Results

### Patient characteristics

Clinical characteristics of the enrolled 117 patients are summarized in Table 1. Among these 117 patients, 90 patients (76.9%) showed increased FDG uptake in the gastric cancer lesion, whereas the remaining 27 patients (23.1%) showed no discernible increase in FDG uptake of the primary tumor lesion. Further, the SUVmaxT of those 17 patients was measured based on contrast-enhanced CT and gastroduodenoscopy findings. At the time of analysis, 42 patients (35.9%) experienced cancer recurrence after curative surgical resection and 23 patients (19.7%) died. The median duration of follow-up was 37.0 months (range 2.6–77.2 months). Of the 42 patients with cancer recurrence, both peritoneal recurrence and distant recurrence were found in 20 patients (47.6%) and locoregional recurrence was found in 10 patients (23.8%). Eight patients (19.0%) had two sites of recurrence at the time of cancer recurrence diagnosis (peritoneal and distant recurrence, 4 patients; locoregional and peritoneal recurrence, 2 patients; locoregional and distant recurrence, 2 patients). Of the 42 patients showing recurrence, 14 patients (33.3% of the patients with recurrence) experienced peritoneal recurrence without locoregional or distant recurrence.

**Table 1:** Characteristics of enrolled patients with advanced gastric cancer (*n* = 117)

Characteristics	No. of patients (%)	Median (range)
Age (years)		63 (34–89)
Gender		
Male	41 (35.0%)	
Female	76 (65.0%)	
Tumor location		
Upper	9 (7.7%)	
Middle	46 (39.3%)	
Lower	60 (51.3%)	
Multiple	2 (1.7%)	
Histopathology		
PAC/TAC	72 (61.6%)	
PDAC	30 (25.6%)	
SRC/mucinous carcinoma	15 (12.8%)	
Lauren classification		
Intestinal	41 (35.0%)	
Non-intestinal	76 (65.0%)	
pT stage		
T2 stage	40 (34.2%)	
T3 stage	46 (39.3%)	
T4 stage	31 (26.5%)	
Lymph node metastasis		
Absence	37 (31.6%)	
Presence	80 (68.4%)	
TNM stage		
Stage I	23 (19.7%)	
Stage II	44 (37.6%)	
Stage III	50 (42.7%)	
Tumor size (cm)		4.9 (1.5–17.0)
Adjuvant treatment		
Yes	78 (66.7%)	
No	39 (33.3%)	
Serum CRP (mg/dL)		2.02 (0.01–78.02)
NLR		2.20 (0.71–8.60)
PLR		141.97 (2.93–425.43)
SUVmaxT		4.85 (2.19–24.60)
VAT attenuation		− 92.38 (− 108.42 to − 73.17)
VAT SUV		0.45 (0.22–0.90)

*PAC* papillary adenocarcinoma, *TAC* tubular adenocarcinoma, *PDAC* poorly differentiated adenocarcinoma, *SRC* signet-ring cell carcinoma, *CRP* C-reactive protein, *NLR* neutrophil-to-lymphocyte ratio, *PLR* platelet-to-lymphocyte ratio, *SUVmaxT* maximum standardized uptake value of primary tumor, *VAT* visceral adipose tissue, *SUV* standardized uptake value

### Correlation analysis

On the assessment of reproducibility of VAT parameters, there was substantial agreement between the two readers for the measurement of VAT attenuation (concordance correlation coefficient: 0.994, 95% confidence interval: 0.990–0.997) and VAT SUV (concordance correlation coefficient: 0.988, 95% confidence interval: 0.979–0.995).

CT attenuation and SUV of VAT showed a significant positive correlation with each other (*p* < 0.001, *r* = 0.799). In correlation analyses of VAT attenuation and SUV with stage, tumor factors, serum inflammatory markers, and clinical outcomes, increased VAT attenuation and SUV were observed in patients with advanced tumors and worse clinical outcomes (Table 2). For T stage and TNM stage, Kruskal–Wallis test showed significant differences of VAT attenuation and SUV between patients with T2, T3, and T4 stages and between patients with stage I, II, and III (*p* < 0.05). On post hoc analysis, patients with T4 stage and stage III had significantly higher VAT attenuation and SUV than those with T2 and T3 stages and those with stage I and II, respectively (*p* < 0.05). Patients with lymph node metastasis also showed significantly higher VAT SUV than those with no lymph node metastasis (*p* = 0.034). For tumor factors, there were significant, but, weak positive correlations between tumor size and both VAT parameters and between SUVmaxT and VAT SUV (*p* < 0.05). In contrast, no significant differences of VAT attenuation and SUV were shown according to the tumor histopathology and Lauren classification (*p* > 0.05). For serum inflammatory markers, only PLR showed significant positive correlations with both VAT attenuation and SUV (*p* < 0.05). The comparisons of VAT attenuation and SUV were also performed between 38 patients who had experienced cancer recurrence at 3-year after surgery and 62 patients who were on regular follow-up at 3-year after surgery with no recurrence, excluding 17 patients who were censored within 3-year after surgery. The results of the comparison analysis revealed that patients with recurrence during the first 3-year after surgery had significantly higher VAT attenuation (*p* = 0.011, − 89.66 ± 9.23 vs. − 94.11 ± 7.73) and VAT SUV (*p* = 0.004, 0.54 ± 0.16 vs. 0.45 ± 0.14) than those with no recurrence during the first 3-year.

**Table 2:** Results of correlation analyses

	VAT attenuation	VAT SUV
T stage		
T2	− 94.93 ± 7.64	0.44 ± 0.14
T3	− 93.96 ± 7.85	0.46 ± 0.12
T4	− 87.28 ± 9.14	0.57 ± 0.18
*p* value	< 0.001	0.007
Lymph node metastasis		
Absence	− 93.98 ± 7.80	0.44 ± 0.13
Presence	− 91.89 ± 9.03	0.50 ± 0.16
*p* value	0.221	0.034
TNM stage		
Stage I	− 95.80 ± 8.27	0.42 ± 0.14
Stage II	− 96.43 ± 6.87	0.42 ± 0.11
Stage III	− 88.10 ± 8.38	0.56 ± 0.16
*p* value	< 0.001	< 0.001
Tumor size		
*r*	0.205	0.228
*p* value	0.027	0.013
SUVmaxT		
*r*	0.158	0.197
*p* value	0.068	0.033
Histopathology		
PAC/TAC	− 92.40 ± 8.91	0.47 ± 0.15
PDAC/SRC/mucinous carcinoma	− 92.72 ± 8.39	0.50 ± 0.16
*p* value	0.846	0.402
Lauren classification		
Intestinal	− 93.18 ± 8.97	0.49 ± .15
Non-intestinal	− 92.16 ± 8.56	0.48 ± 0.15
*p* value	0.547	0.793
Serum CRP		
*r*	0.012	0.080
*p* value	0.898	0.391
NLR		
*r*	0.122	0.164
*p* value	0.192	0.077
PLR		
*r*	0.305	0.318
*p* value	0.008	0.005

*SUVmaxT* maximum standardized uptake value of primary tumor, *PAC* papillary adenocarcinoma, *TAC* tubular adenocarcinoma, *PDAC* poorly differentiated adenocarcinoma, *SRC* signet-ring cell carcinoma, *CRP* C-reactive protein, *NLR* neutrophil-to-lymphocyte ratio, *PLR* platelet-to-lymphocyte ratio, *r* Pearson’s correlation coefficient, *VAT* visceral adipose tissue, *SUV* standardized uptake value

### Survival analysis

The prognostic values of VAT attenuation and SUV for predicting RFS, peritoneal RFS, and OS were assessed along with age, sex, tumor factors, serum inflammatory markers, and SUVmaxT. For continuous variables, optimal cut-off values were selected by the ROC curve analysis, which were 60 years for age, 5.0 cm for tumor size, 5.00 mg/dL for serum C-reactive protein (CRP) level, 2.30 for NLR, 173.76 for PLR, 4.65 for SUVmaxT, − 90.60 for VAT attenuation, and 0.46 for VAT SUV. ROC curve analysis revealed that the sensitivity, specificity, positive predictive value, and negative predictive value of VAT attenuation were 54.8%, 70.7%, 51.1%, and 73.6% for recurrence; 60.0%, 67.0%, 27.2%, and 89.0% for peritoneal recurrence; and 78.3%, 73.4%, 41.9%, and 93.2% for death, using a cut-off value of − 90.60. The sensitivity, specificity, positive predictive value, and negative predictive value of VAT SUV were 83.3%, 38.7%, 43.2%, and 80.6% for recurrence; 70.0%, 58.8%, 25.9%, and 90.5% for peritoneal recurrence; and 91.3%, 63.8%, 38.2%, and 96.8% for death, using a cut-off value of 0.46.

**Fig. 2:**
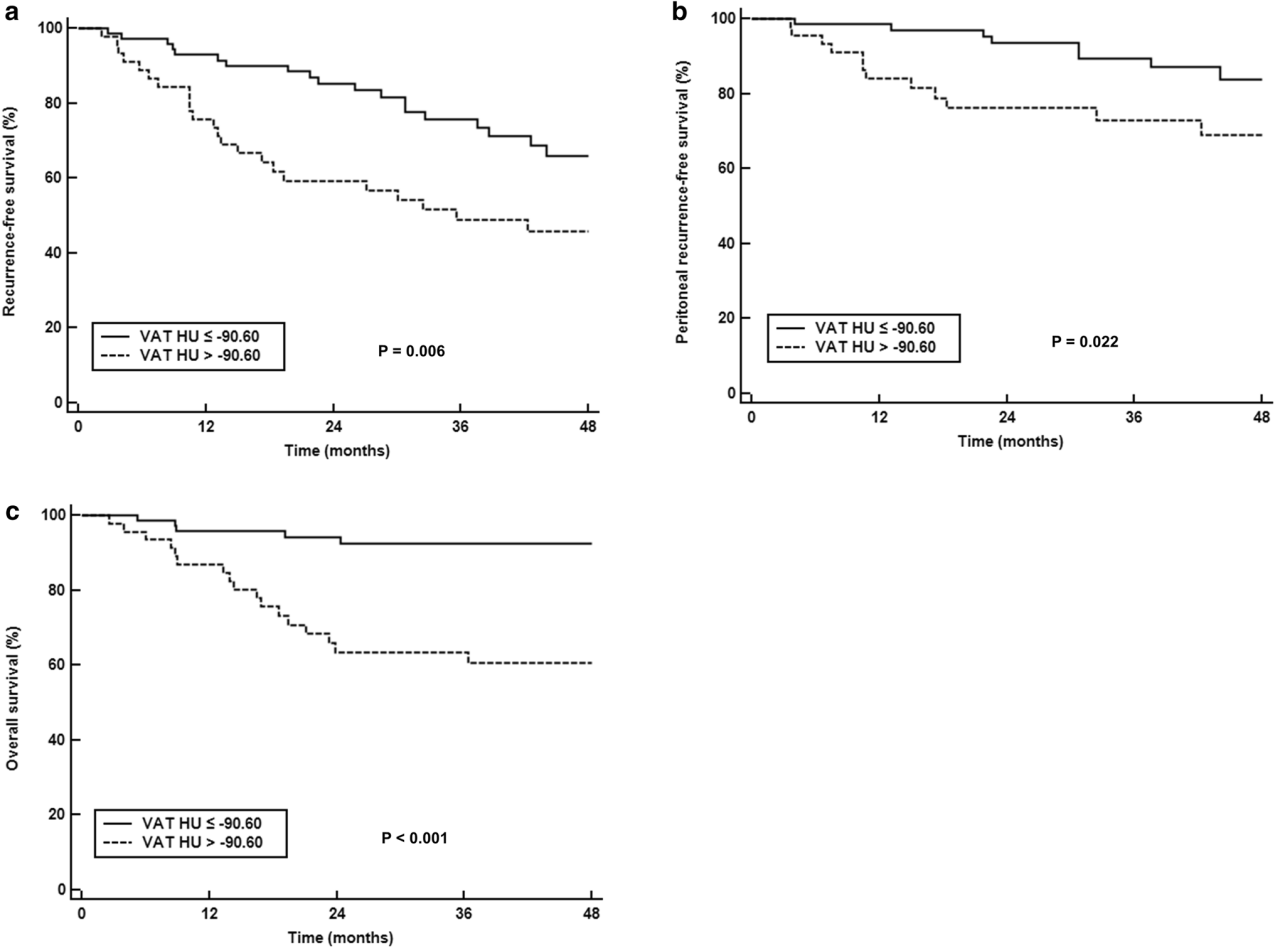
Kaplan-Meier RFS (**a**), peritoneal RFS (**b**), and OS (**c**) curves according to CT attenuation of VAT

**Fig. 3:**
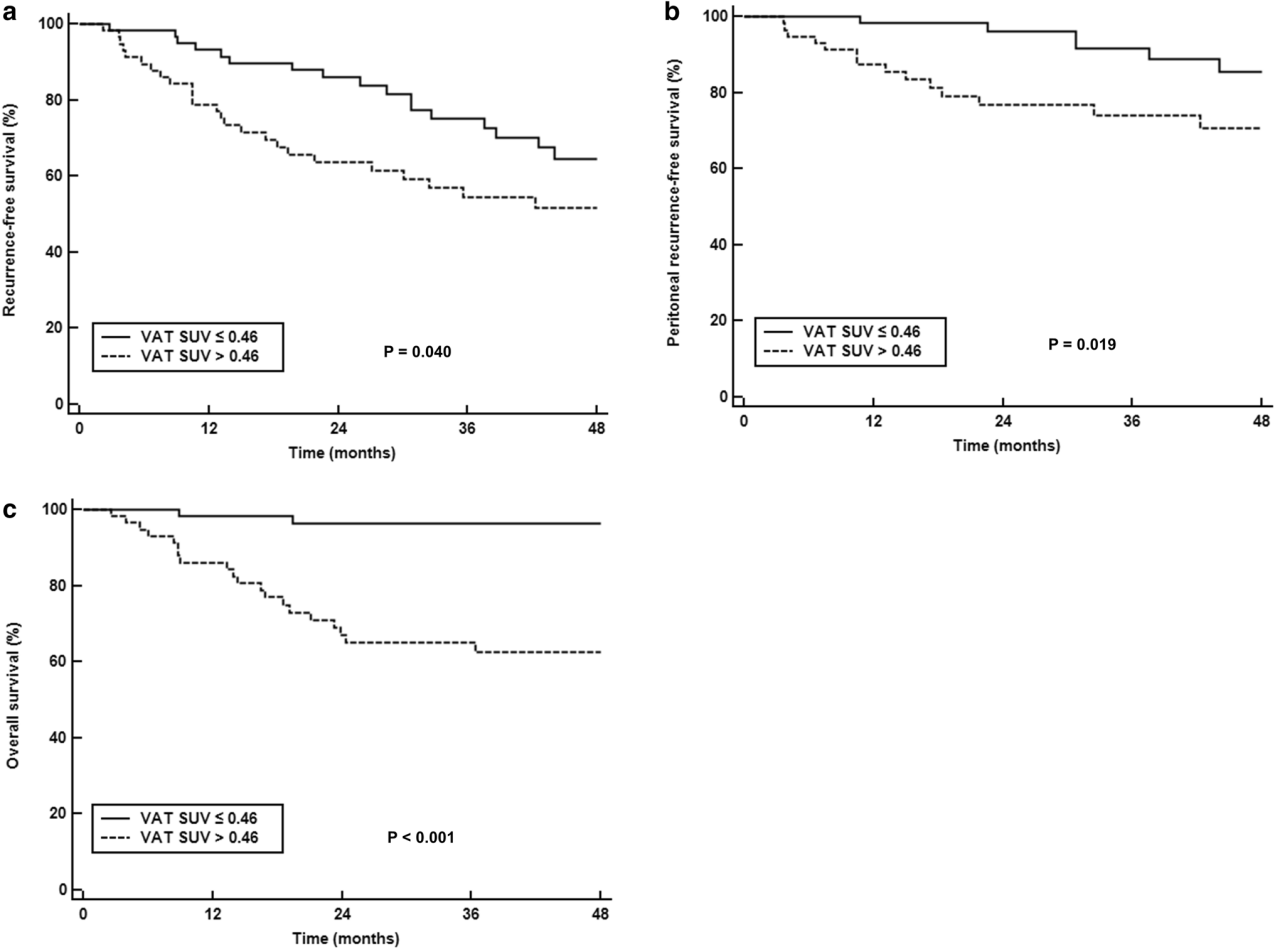
Kaplan-Meier RFS (**a**), peritoneal RFS (**b**), and OS (**c**) curves according to FDG uptake of VAT

On univariate survival analysis, both VAT attenuation and SUV were significantly associated with RFS, peritoneal RFS, and OS (*p* < 0.05; Table 3). Patients with high VAT attenuation and SUV had significantly worse survival than those with low values (Figs. 2 and 3). In addition to VAT attenuation and SUV, T stage, presence of lymph node metastasis, tumor size, and NLR were significant predictors for RFS, peritoneal RFS, and OS (*p* < 0.05). SUVmaxT and PLR were associated with only RFS and OS, and tumor histopathology was a significant predictor for only peritoneal RFS (*p* < 0.05).

**Table 3:** Univariate analyses for RFS, peritoneal RFS, and OS

Variables	RFS		Peritoneal RFS		OS	
	*p* value	Hazard ratio (95% CI)	*p* value	Hazard ratio (95% CI)	*p* value	Hazard ratio (95% CI)
Age (≤ 60 vs. > 60)	0.563	1.20 (0.65–2.22)	0.971	1.02 (0.42–2.45)	0.507	1.33 (0.57–3.07)
Sex (female vs. male)	0.402	1.31 (0.70–2.47)	0.139	1.95 (0.80–4.74)	0.741	1.16 (0.49–2.73)
Histopathology (PAC/TAC vs. PDAC/SRC/mucinous carcinoma)	0.323	1.36 (0.74–2.49)	0.045	2.50 (1.02–6.11)	0.940	1.03 (0.45–2.39)
Lauren classification (intestinal vs. non-intestinal)	0.490	1.26 (0.65–2.42)	0.268	1.77 (0.64–4.88)	0.306	1.63 (0.64–4.13)
T stage						
T2		1.00		1.00		1.00
T3	0.010	4.17 (1.40–12.39)	0.043	8.47 (1.07–66.87)	0.103	3.63 (0.77–17.09)
T4	< 0.001	10.27 (3.51–30.04)	0.006	18.16 (2.32–142.30)	0.002	10.64 (2.40–47.23)
Lymph node metastasis (absence vs. presence)	< 0.001	6.38 (2.27–17.93)	0.021	5.61 (1.30–24.28)	0.017	5.87 (1.37–25.08)
Tumor size (≤ 5.0 vs. > 5.0)	0.007	3.10 (1.61–5.97)	0.007	4.03 (1.46–11.21)	0.003	4.49 (1.66–12.10)
Serum CRP (≤ 5.00 vs. > 5.00)	0.223	1.49 (0.78–2.83)	0.170	1.87 (0.76–4.58)	0.167	1.81 (0.78–4.17)
NLR (≤ 2.30 vs. > 2.30)	0.002	2.70 (1.44–5.05)	0.008	3.66 (1.40–9.55)	0.008	3.32 (1.36–8.08)
PLR (≤ 173.76 vs. > 173.76)	0.002	2.68 (1.46–4.93)	0.072	2.25 (0.93–5.46)	0.005	3.28 (1.44–7.50)
SUVmaxT (≤ 4.65 vs. > 4.65)	0.004	2.61 (1.35–5.04)	0.052	2.60 (0.99–6.79)	0.002	6.85 (2.03–23.12)
VAT attenuation (≤ − 90.60 vs. > − 90.60)	0.008	2.28 (1.24–4.20)	0.019	2.72 (1.11–6.66)	< 0.001	6.33 (2.35–17.07)
VAT SUV (≤ 0.46 vs. > 0.46)	0.040	1.88 (1.03–3.49)	0.017	2.99 (1.15–7.78)	< 0.001	12.84 (3.01–54.80)

*RFS* recurrence-free survival, *OS* overall survival, *PAC* papillary adenocarcinoma, *TAC* tubular adenocarcinoma, *PDAC* poorly differentiated adenocarcinoma, *SRC* signet-ring cell carcinoma, *CRP* C-reactive protein, *NLR* neutrophil-to-lymphocyte ratio, *PLR* platelet-to-lymphocyte ratio, *SUVmaxT* maximum standardized uptake value of primary tumor, *VAT* visceral adipose tissue, *SUV* standardized uptake value, *CI* confidence interval

The variables that showed statistical significance (*p* value of < 0.05) for predicting RFS, peritoneal RFS, and OS on univariate analysis were selected for multivariate analysis (Table 4). Bonferroni correction for multiple testing was performed for the multivariate analysis, and *p* value of less than 0.0083 was considered as statistically significant. VAT attenuation and SUV were evaluated on separate models, because both were significantly associated with each other. For serum inflammatory markers, only NLR was included in the analysis, because NLR and PLR showed significant association with each other. VAT attenuation remained as significant positive predictors for peritoneal RFS (*p* = 0.008) and OS (*p* = 0.007) and VAT SUV showed significant association only with OS (*p* = 0.004). Along with VAT attenuation and SUV, tumor histopathology (*p* = 0.008) was an independent predictor for peritoneal RFS and T stage (*p* = 0.006) was an independent predictor for OS. In contrast to peritoneal RFS and OS, both VAT attenuation and SUV failed to show statistical significance for predicting RFS (*p* > 0.0083) on multivariate analysis, and only T stage showed significant association with RFS (*p* < 0.0083).

**Table 4:** Multivariate analyses for RFS, peritoneal RFS, and OS

Variables	RFS				Peritoneal RFS				OS			
	Model with attenuation		Model with SUV		Model with attenuation		Model with SUV		Model with attenuation		Model with SUV	
	*p* value	Hazard ratio (95% CI)	*p* value	Hazard ratio (95% CI)	*p* value	Hazard ratio (95% CI)	*p* value	Hazard ratio (95% CI)	*p* value	Hazard ratio (95% CI)	*p* value	Hazard ratio (95% CI)
Histopathology	–	–	–	–	0.008	3.00 (1.25–7.46)	0.010	3.19 (1.21–8.45)	–	–	–	–
T stage												
T2		1.00		1.00		1.00		1.00		1.00		1.00
T3	0.035	3.29 (1.09–9.34)	0.037	3.25 (1.08–9.80)	0.135	4.98 (0.61–40.78)	0.063	7.16 (0.90–57.09)	0.158	5.27 (0.65–14.49)	0.312	2.29 (0.46–11.30)
T4	0.005	5.08 (1.66–15.57)	0.003	5.32 (1.73–16.14)	0.025	10.90 (1.35–88.16)	0.026	10.88 (1.32–89.38)	0.006	5.27 (1.24–24.32)	0.010	3.36 (1.15–17.20)
Lymph node metastasis	0.038	3.20 (1.07–9.59)	0.030	3.35 (1.12–10.02)	0.159	3.06 (0.65–14.52)	0.200	2.83 (0.58–13.84)	0.527	1.66 (0.35–7.99)	0.511	1.70 (0.35–8.30)
Tumor size	0.295	1.46 (0.72–2.99)	0.167	1.62 (0.82–3.23)	0.033	3.25 (1.10–9.63)	0.049	3.05 (3.05–9.27)	0.452	1.54 (0.50–4.78)	0.311	1.82 (0.57–5.78)
NLR	0.053	1.93 (0.99–3.79)	0.015	2.21 (1.16–4.19)	0.063	2.68 (0.95–7.57)	0.101	2.28 (0.85–6.09)	0.251	1.72 (0.68–4.32)	0.175	1.89 (0.75–4.77)
SUVmaxT	0.015	2.21 (1.16–4.19)	0.039	2.01 (1.04–3.89)	–	–	–	–	0.012	4.28 (1.24–14.81)	0.139	2.66 (0.73–9.74)
VAT attenuation	0.477	1.28 (0.65–2.53)	–	–	0.008	3.86 (1.47–1.12)	–	–	0.007	3.83 (1.36–10.87)	–	–
VAT SUV	–	–	0.530	1.23 (0.64–2.36)	–	–	0.019	2.99 (1.20–7.48)	–	–	0.004	8.45 (1.97–37.02)

Statistically significant for variables with *p* < 0.0083 after Bonferroni adjustment

*RFS* recurrence-free survival, *OS* overall survival, *NLR* neutrophil-to-lymphocyte ratio, *PLR* platelet-to-lymphocyte ratio, *SUVmaxT* maximum standardized uptake value of primary tumor, *VAT* visceral adipose tissue, *SUV* standardized uptake value, *CI* confidence interval

## Discussion

In the present study, CT attenuation and FDG uptake of VAT, which represent qualitative characteristics of VAT, showed significant positive association with peritoneal recurrence and mortality risk. Recently, several studies have demonstrated a significant link between adipocytes and cancer cells [8, 10]. Dysfunctional adipocytes in pathological conditions such as obesity secrete multiple proinflammatory cytokines and angiogenic factors, causing chronic inflammation with the infiltration of immune cells, which is a favorable microenvironment for carcinogenesis and tumor cell growth [8]. Furthermore, upon exposure to cancer cells, adipocytes are modified by losing intracellular lipid content, upregulating secretion of inflammatory cytokines, and exhibiting fibroblast-like morphology [20]. These modified adipocytes, the so-called cancer-associated adipocytes, conversely further promote tumor progression and metastasis [21]. The link between adipocytes and tumor cells also appears in gastric cancer cells. A previous study with an in vitro co-culture system revealed that omental adipocytes supplied oleic acid to gastric cancer cells and promoted cancer cell invasion, which might be one of the reasons of predilection of gastric cancer cells for peritoneal metastasis [16].

At present, two imaging parameters, CT attenuation and FDG uptake, have been developed to estimate qualitative changes of the adipose tissue microenvironment [12]. A biopsy study with non-human primates revealed that high CT attenuation of adipose tissue corresponded to smaller adipocytes with low intracellular lipid content and increased extracellular matrix fibrosis, which are also findings in the adipose tissue microenvironment of cancer-associated adipocytes [10, 11, 14]. Clinical studies with pancreatic cancer, extremity sarcoma, head and neck cancer, and prostate cancer have consistently demonstrated that patients with high CT attenuation of adipose tissue had significantly worse clinical outcomes than those with low attenuation, implying the significant association between qualitative changes of adipose tissue reflected on CT attenuation and tumor progression [12, 14, 22, 23]. FDG uptake of adipose tissue is known to reflect glucose metabolism of adipocytes and inflammatory cells recruited in the adipose tissue [13, 19, 24, 25]. Although previous studies have found a significant relationship between FDG uptake of adipose tissue and tumor factors, the results are contradictory between these studies [12, 13, 24, 25]. A previous study with pancreatic cancer patients showed that patients with lymph node or distant metastases had significantly lower FDG uptake of subcutaneous adipose tissue than those with no metastasis, postulating that cancer cells in advanced stage tend to inhibit adipocyte glucose metabolism to use more exogenous fatty acids for tumor cell growth [24]. In contrast, other studies with colon cancer patients and another study with pancreatic cancer patients revealed that advanced stage patients had significantly higher FDG uptake of VAT and FDG uptake of VAT was an independent predictor for survival [13, 19, 25]. Authors in these studies suggested that FDG uptake of VAT is related to the macrophage-induced inflammatory response in VAT [13, 25]. Considering that cancer-associated adipocytes can recruit and modulate macrophages and both cancer-associated adipocytes and macrophages play important roles in tumor microenvironment [26], FDG uptake of VAT might indirectly reflect interaction between adipocytes and cancer cells and have significant associations with tumor aggressiveness and clinical outcomes [12, 25].

In the present study, CT attenuation of VAT showed significant association with peritoneal RFS and OS even after adjusting clnico-histologic factors. Furthermore, there was a significant positive correlation between CT attenuation and FDG uptake of VAT and VAT SUV showed significant association with OS. It implies that FDG uptake of VAT mainly reflects inflammatory and fibrotic changes in VAT and both VAT attenuation and SUV can be used as imaging biomarkers for qualitative changes of VAT in patients with AGC. As shown in previous studies with extremity sarcoma, prostate cancer, colorectal cancer and pancreatic cancer [12, 14, 22, 25], patients with gastric cancer also demonstrated significant association between qualitative features of VAT and survival. This association in our study mainly results from the aforementioned link between adipocytes and cancer cells [8, 10]. In our study, patients with T4 stage had significantly higher VAT attenuation and SUV than those with T2 and T3 stages, which support this explanation. Because T4 stage gastric cancer invades visceral peritoneum [17], there is a direct contact between VAT adipocytes and gastric cancer cells, which could cause profound interaction between them [10]. Furthermore, a significant positive correlation between SUVmaxT and VAT SUV suggests that qualitative alterations of VAT might be more pronounced in tumors with aggressive features. It has been already known that the success of cancer cell implantation in the distant organ is determined by not only metastatic competence of cancer cells but also a permissive microenvironment of the organ, which is called the “seed and soil” theory [27]. Therefore, increased CT attenuation and FDG uptake of VAT might represent a VAT microenvironment in which gastric cancer cells could easily recurred and progressed. Patients with high VAT attenuation can be considered as having high risk of peritoneal recurrence and might be good candidates for adjuvant intraperitoneal chemotherapy after surgery [4]. However, considering the low positive predictive values of VAT attenuation and SUV for recurrence and peritoneal recurrence, a prediction model for recurrence consisted of VAT parameters and tumor factors should be designed in future researches rather than using VAT attenuation and SUV alone.

The second possible explanation for the findings in our study is hidden metastatic cancer cells in the peritoneum. In patient enrollment, we excluded patients who showed peritoneal seeding metastases or malignant cells on peritoneal washing cytology. However, enrolled patients might possess hidden micro-metastasis in the peritoneum, which would affect VAT attenuation and SUV and lead to worse survival after surgery [25].

In correlation analyses with serum inflammatory markers, none of the adipose tissue parameters showed significant relationship with serum CRP and NLR. Previous studies have already shown the insignificant relationship of adipose tissue parameters with serum CRP and NLR, concluding that serum inflammatory markers had limited values for predicting alterations of adipose tissue [11, 12]. In contrast, both VAT attenuation and SUV showed significant positive correlation with PLR. Activated platelets are known to promote tumor growth and metastasis and, in previous studies with gastric cancer patients, PLR showed significant association with the presence of lymph node metastasis and OS [28–30]. Moreover, although only borderline significance was shown in univariate analysis of our study, a recent study demonstrated that PLR was an independent predictor for peritoneal RFS [31]. Therefore, it could be speculated that activated platelets might make a significant contribution to inflammatory and fibrotic changes of VAT; however, further studies are warranted to discover underlying mechanisms of the relationship.

The present study had some limitations that should not be neglected. First, because the present study was retrospectively performed in a single hospital, further validation is required by multicenter studies with a large number of patients. Second, the site of cancer recurrence was determined mainly by imaging examinations; thus, there might be possibility that exact sites of recurrence were underestimated in some of the enrolled patients. Finally, because of a small number of studies regarding qualitative features of adipose tissue, there is no consensus on calculating CT attenuation and FDG uptake of adipose tissue, which limits comparisons with other studies [11, 12, 14, 19, 22, 24].

In conclusion, CT attenuation of VAT were independently associated with peritoneal RFS and OS and FDG uptake VAT was an independent predictor for OS. Both VAT attenuation and SUV had significant positive associations with T stage, tumor size, and PLR. In survival analysis, patients with high VAT attenuation and SUV had worse RFS, peritoneal RFS and OS than those with low values. Further studies are warranted to validate the results of the study.
